# Supplementary material for: Jekyll or Hyde? The genome (and more) of Nesidiocoris tenuis, a zoophytophagous predatory bug that is both a biological control agent and a pest
Source: Insect Mol Biol. 2020 Dec 22;30(2):188–209. doi: 10.1111/imb.12688 (PMC8048687; doi:10.1111/imb.12688)
Supplement: Supplementary file 1 — Appendix S1: Supporting Information. [file IMB-30-188-s001.docx]

*Nesidiocoris tenuis* genome – Supplementary information S1

Table of Contents

[S1.1. *Wolbachia* PCR results 2](#_Toc48922888)

[Figure S1.1.1 2](#_Toc48922889)

[S1.2 BUSCO details 3](#_Toc48922890)

[S1.3. Flow cytometry data 4](#_Toc48922891)

[S1.4. Decontamination and potential LGT identification 5](#_Toc48922892)

[S1.5. Full protein set 6](#_Toc48922893)

[S1.6. Cytogenetics: Southern blot, abundance distributions, and primers 7](#_Toc48922894)

[Figure S1.6.1. 7](#_Toc48922895)

[Figure S1.6.2. 8](#_Toc48922896)

[Figure S1.6.3. 9](#_Toc48922897)

[S1.7. Poolseq results in full 9](#_Toc48922898)

[References 10](#_Toc48922899)

For DANS EASY Repository data:

Pannebakker, dr. ir. B. A. (Wageningen University); Ferguson, K. B. (Wageningen University) (2019): The *Nesidiocoris tenuis* genome manuscript supporting data. DANS. https://doi.org/10.17026/dans-z5z-zec9

# S1.1. *Wolbachia* PCR results





**1 2 3 4 5 6 7**

### Figure S1.1.1

*Wolbachia* detection was carried out according to the PCR protocol of Zhou et al. (Zhou, Rousset, and O’Neill, 1998). Lane 1 contains a 100 bp ladder, while lanes 2 and 3 contain PCR products for *Nesidiocoris tenuis* DNA (see 18S procedure). Lanes 4 and 5 contain sterilized water as a negative control, while lanes 6 and 7 contain PCR products for *Ephestia kuehniella* DNA (see Southern Blot procedure).

# S1.2 BUSCO details

Files on figshare: <https://figshare.com/articles/dataset/Nesidiocoris_tenuis_linked-read_genome_supplementary_materials_BUSCO_scaffold_comparisons/12841403>

DOI:10.6084/m9.figshare.12841403

S1.2.1 BUSCO full table results (direct output from BUSCO software (Simão *et al.*, 2015))

S1.2.2 Scaffolds ≥95% identity with e=100 (using *dedupe* tool within bbtools (BBMap – Bushnell B. – sourceforge.net/projects/bbmap/)

S1.2.3 Comparisons between both outputs

# S1.3. Flow cytometry data

Files associated with the flow cytometry data collected to determine genome size. The .FCS files of the results include: two propidium-iodide stained (PI-stained) *Nesidiocoris tenuis* samples, one sample of PI-stained *N. tenuis* dosed with head of *Drosophila melanogaster*, one unstained *N. tenuis* background sample, and three PI-stained *D. melanogaster* samples. Available on DANS in collection, refer to these files:

- original/20160226_26_Nesidiocoristenuis1.fcs
- original/20160226_27_Nesidiocoristenuis2.fcs
- original/20160226_28_NesidiocoristenuisDMelanogaster.fcs
- original/20160226_11_unstained_Nesidiocoristenuis.fcs
- original/20160226_Dmelanogaster_PI stained_3.fcs
- original/20160226_DMelanogaster_PI stained_4.fcs
- original/20160226_DMelanogaster_PI stained_5.fcs

# S1.4. Decontamination and potential LGT identification

S1.4.1 In-house reference bacterial database that contains 2,100 different bacterial species. Adapted from (Wheeler, Redding, and Werren, 2013). Available on DANS:

- original/S1.3.1_LGTPipeline_BacteriaSet.csv

S1.4.2 List of bacterial contamination, identified an adapted pipeline (Wheeler, Redding, and Werren, 2013). Scaffold numbers correspond to removed scaffolds in S1.4.3. Available on DANS:

- original/S1.3.2_Nesidiocoris_BacterialScaffolds.txt

S1.4.3 The .FASTA files of scaffolds deemed contamination. Note, the names are no longer valid when compared to the current assembly. Available on DANS:

- original/S1.3.3_Contamination.fa

S1.4.4 List of potential LGT candidates, limited selection, available on DANS:

- original/S1.3.4_Nesidiocoris_LGTsManual_Limited.csv

# S1.5. Full protein set

The .faa (amino acid .fasta) used in the protein cluster analysis is the official protein set of the *Nesidiocoris tenuis* genome and is available on DANS.

- original/Ntenuis_protein.aa.fasta

# S1.6. Cytogenetics: Southern blot, abundance distributions, and primers


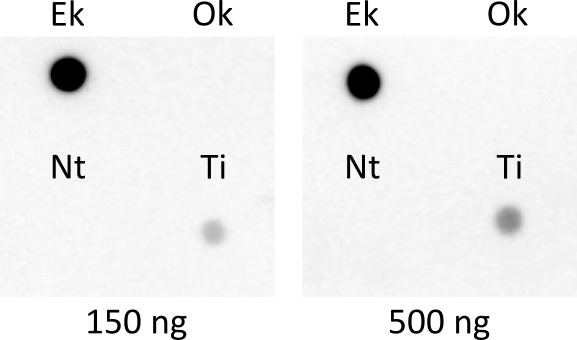


## Figure S1.6.1.

Southern dot blot assay using the insect telomere motif (TTAGG)*_n_* in *Ephestia kuehniella*, *Oncorhynchus keta*, *Nesidiocoris tenuis*, and *Triatoma infestans*. *E. kuehniella* (Ek) and *T. infestans* (Ti) show hybridization signals, whilst *N. tenuis* (Nt) and the negative control *O. keta* (Ok) display no detectable hybridization signals. Both quantities of DNA (150 ng and 500 ng) show comparable results.

| Table S1.6.1. Abundance and distribution of Nt_rep1 in the assembled genome | | |
| --- | --- | --- |
| **# of Nt_rep1 copies per scaffold** | **Number of scaffolds in the assembly** | **% of total** |
| 1 | 2625 | 78.45 |
| 2 | 441 | 13.18 |
| 3 | 148 | 4.42 |
| 4 | 74 | 2.21 |
| 5 | 32 | 0.96 |
| 6 | 11 | 0.33 |
| 7 | 3 | 0.09 |
| 8 | 6 | 0.18 |
| 9 | 3 | 0.09 |
| 11 | 2 | 0.06 |
| 17 | 1 | 0.03 |


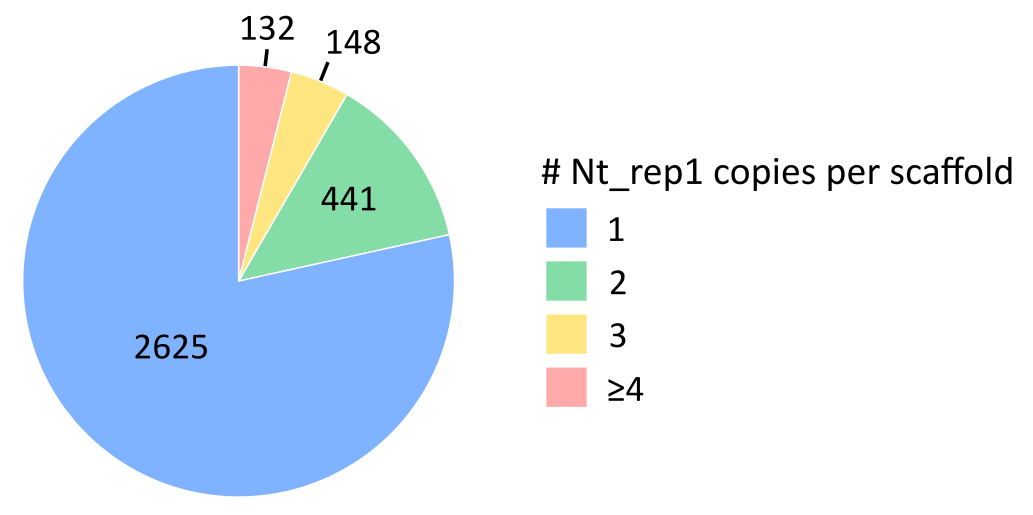


## Figure S1.6.2.

Distribution of Nt_rep1 on the assembled scaffolds. Note that most of the scaffolds containing Nt_rep1 carry only a single copy.

| Table S1.6.2. Overview of cytogenetic primers used in this study. | | | |
| --- | --- | --- | --- |
| **Name** | **Sequence 5’-3’** | **Function** | **Reference** |
| 18S-1 | CTG GTT GAT CCT GCC AGT AGT | 18S rDNA partial | (Jung and Lee, 2012) |
| 18S-4 | GAT CCT TCT GCA GGT TCA CC | 18S rDNA partial | (Jung and Lee, 2012) |
| “Ins_telo_F” | TAG GTT AGG TTA GGT TAG GT | Insect telomere motif | (Sahara, Marec, and Traut, 1999) |
| “Ins_telo_R” | CTA ACC TAA CCT AAC CTA AC | Insect telomere motif | (Sahara, Marec, and Traut, 1999) |
| Nt_pt1_F | ATG GTA TGG TAT GGT ATG GT | *N. tenuis* potential telomere motif 1 | This study |
| Nt_pt1_R | CAT ACC ATA CCA TAC CAT AC | *N. tenuis* potential telomere motif 1 | This study |
| Nt_pt2_F | TGG GTT GGG TTG GGT TGG GT | *N. tenuis* potential telomere motif 2 | This study |
| Nt_pt2_R | CCA ACC CAA CCC AAC CCA AC | *N. tenuis* potential telomere motif 2 | This study |
| Nt_pt3_F | CAG GTC AGG TCA GGT CAG GT | *N. tenuis* potential telomere motif 3 | This study |
| Nt_pt3_R | CTG ACC TGA CCT GAC CTG AC | *N. tenuis* potential telomere motif 3 | This study |
| Nt_rep1_F | TTC GCC CAA AAT GAA AAA ACG C | Nt_rep1 | This study |
| Nt_rep1_R | TCC TGA ACA AGT GTC TGT GTG T | Nt_rep1 | This study |

## Figure S1.6.3.


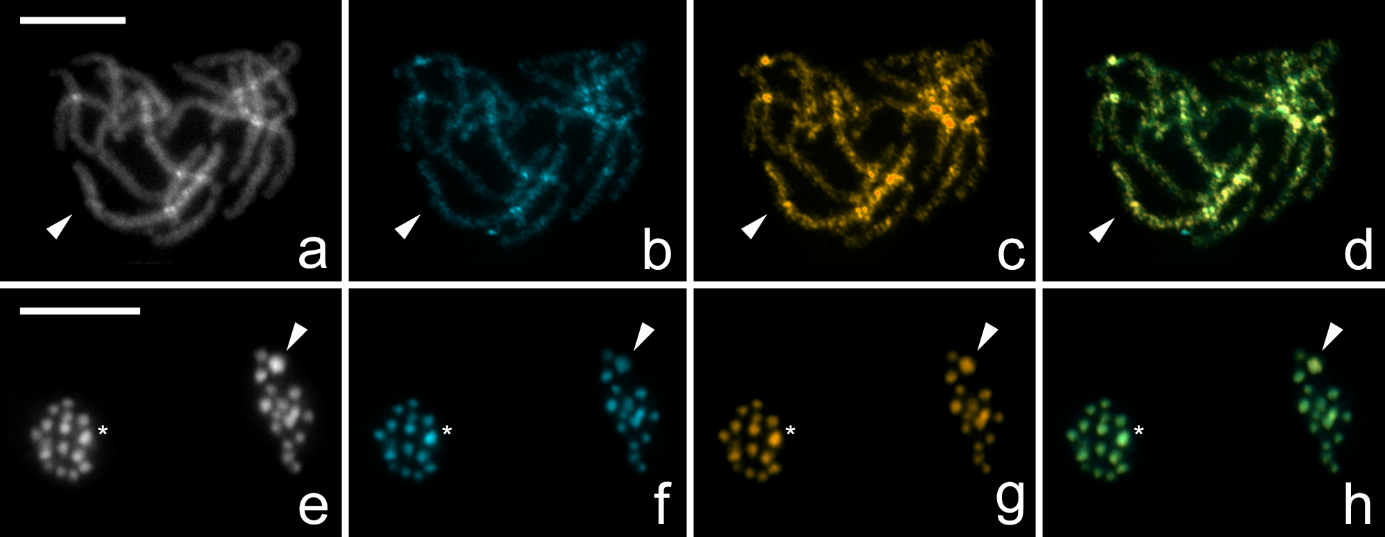


Alternate colouration for Figure 5 in-text. Comparative genomic hybridization (CGH) on female pachytene (**a**, **b**, **c**, **d**) and male meiotic metaphase II (**e**, **f**, **g**, **h**) chromosomes of *Nesidiocoris tenuis*. Panels (**a**, **e**) show chromosomes counterstained with DAPI (grey), panels (**b**, **f**) hybridization signals of the male derived genomic probe labelled by fluorescein (blue), panels (**c**, **g**) hybridization signals of the female derived genomic probe labelled by Cy3 (gold), and panels (**d**, **h**) merged images. (**c**, **d**) Note that the X chromosome bivalent (arrowhead) in female pachytene complement was highlighted more by female probe compared to the autosomal bivalents; (**b**, **d**) male probe labelled all chromosomes equally. (**h**) Two sister nuclei in meiotic metaphase II showed equal hybridization patterns of both probes on autosomes; in one of the forming nuclei, the X chromosome (arrowhead) was highlighted by female derived genomic probe (**g**, **h**) and in the second nucleus the Y chromosome (asterisk) was strongly highlighted by male derived genomic probe compared to autosomes (**f**, **h**) and less highlighted by female derived probe (**g**, **h**). (**e**) Note that the sex chromosomes are the biggest and most heterochromatic elements in the nucleus. Scale bar = 10 µm.

# S1.7. Poolseq results in full

Full results (*Tajima’s D* and *Tajima’s π*) of PoPoolation runs, under three conditions:

S1.9.1 Sliding window and step size 10K,

- original/S1.9.1 PoPoolationResults.csv

S1.9.2 Sliding window and step size 5K,

- original/S1.9.2 PoPoolationResults.csv

S1.9.3 Indel masking with sliding window and step size 10K (reported results).

- original/S1.9.3 PoPoolationResults_ReportedResults.csv

# References

Jung, S. and Lee, S. (2012), “Molecular phylogeny of the plant bugs (Heteroptera: Miridae) and the evolution of feeding habits”, *Cladistics*, Vol. 28 No. 1, pp. 50–79.

Sahara, K., Marec, F. and Traut, W. (1999), “TTAGG telomeric repeats in chromosomes of some insects and other arthropods”, *Chromosome Research*, Vol. 7 No. 6, pp. 449–460.

Simão, F.A., Waterhouse, R.M., Ioannidis, P., Kriventseva, E. V. and Zdobnov, E.M. (2015), “BUSCO: Assessing genome assembly and annotation completeness with single-copy orthologs”, *Bioinformatics*, Vol. 31 No. 19, pp. 3210–3212.

Wheeler, D., Redding, A.J. and Werren, J.H. (2013), “Characterization of an Ancient Lepidopteran Lateral Gene Transfer”, *PLoS ONE*, Vol. 8 No. 3, available at:https://doi.org/10.1371/journal.pone.0059262.

Zhou, W., Rousset, F. and O’Neill, S. (1998), “Phylogeny and PCR-based classification of Wolbachia strains using wsp gene sequences”, *Proceedings of the Royal Society B: Biological Sciences*, Vol. 265 No. 1395, pp. 509–515.
